# Fatal Rat-Bite Fever in a Child — San Diego County, California, 2013

**Published:** 2014-12-19

**Authors:** Jessica K. Adam, Aiden K. Varan, Alice L. Pong, Eric C. McDonald

**Affiliations:** 1 Epidemic Intelligence Service, CDC; 2 County of San Diego Health and Human Services Agency, San Diego, California; 3 Division of Global Migration and Quarantine, National Center for Emerging and Zoonotic Infectious Diseases, CDC; 4 CDC and Council of State and Territorial Epidemiologists Applied Epidemiology Fellowship; 5 Rady Children’s Hospital, San Diego, California; 6 Division of Pediatric Infectious Diseases, University of California San Diego, San Diego, California

In August 2013, the County of San Diego Health and Human Services Agency was notified of a fatal case of rat-bite fever (RBF) in a previously healthy male, aged 10 years, who owned pet rats. Two days before his death, the patient experienced rigors, fevers, vomiting, headaches, and leg pains. His physician noted a fever of 102.6°F (39.2ºC), documented a normal examination, diagnosed viral gastroenteritis, and prescribed anti-nausea medication. During the next 24 hours, the patient experienced vomiting and persistent fever. He was confused and weak before collapsing at home. Paramedics reported the patient was unresponsive and had dilated pupils; resuscitation was initiated in the field and was continued for >1 hour after arrival at the emergency department but was unsuccessful. A complete blood count performed during resuscitation revealed anemia (hemoglobin 10.0 g/dL [normal = 13.5–18.0 g/dL], thrombocytopenia (platelets 40,000/*μ*L [normal = 140,000–440,000/*μ*L]), leukocytosis (white blood cells 17,900 cells/*μ*L [normal = 4,000–10,500/*μ*L]) with 16% band neutrophils; the patient also had evidence of disseminated intravascular coagulation. No rash or skin breakdown was noted. Lung, liver, and epiglottis tissue collected postmortem was positive for *Streptobacillus moniliformis* DNA by polymerase chain reaction.

During the 10 days before his death, the patient had obtained his second pet rat; *S. moniliformis* was detected by polymerase chain reaction in oropharyngeal tissue from this rat. Oropharyngeal swabs of the first pet rat were negative for *S. moniliformis* by polymerase chain reaction. The autopsy report noted that patient had been scratched by his pet rats.

RBF is a systemic illness of humans caused principally by *S. moniliformis*, a gram-negative bacterium that is commensal among rats (1). The organism can be transmitted to humans through rodent bites or scratches; approximately one in 10 bites might cause infection (2). Infection can also occur after handling infected rodents without a bite or scratch, or through ingestion of food or water contaminated with the bacteria (1). Symptoms include fever, rash, vomiting, and muscle or joint pain. RBF is treatable with antibiotics (3); approximately 13% of untreated RBF illnesses are fatal (2).

Nearly all domestic and wild rats carry *S. moniliformis* (2). An estimated 0.1% of U.S. households owned one or more pet rats during 2011 (Sharon Granskog, American Veterinary Medical Association, personal communication, April 25, 2014).

RBF is not a reportable condition in California or nationally. To estimate RBF incidence in San Diego County, hospitals in San Diego County that discharged any patients during 2000–2012 with *International Classification of Diseases, Ninth Revision* codes 026.0–026.1 (for streptobacillary fever and spirillary fever) were identified based on data from the California Office of Statewide Health Planning and Development. Medical records were requested, and 16 cases were identified. One additional RBF case was reported to the County of San Diego Health and Human Services Agency during 2013 as an occurrence of unusual disease.

Among the 17 cases, the median patient age was 10 years (range = 4–67 years); 59% of patients were female, and 65% were healthy before infection. Most infections (94%) were pet-associated; one patient had an occupational exposure (rat breeder). Sixteen of 17 patients reported exposure to rats. Of these, 44% reported only having handled a rat, 38% reported being bitten, and 13% reported a scratch. All patients had blood drawn for cultures; only 29% tested positive for *S. moniliformis*; the remainder were treated presumptively for RBF on the basis of exposure and clinical presentation. All patients survived except the patient described in this report.

RBF is a rare but potentially fatal illness that should be considered in persons with rash, fever, and joint pain and when a history of rodent exposure is reported. Clinicians suspecting *S. moniliformis* infection should promptly alert laboratory staff because microbiologic diagnosis is difficult, requiring specific media and incubation conditions. Clinicians should also consider requesting diagnosis assistance from their state public health laboratories. Because rapid laboratory confirmation might not be possible, empiric treatment for RBF in the setting of appropriate exposure history might be considered.

Pet rat owners should wear gloves and wash their hands thoroughly after handling rats or cleaning rat cages, avoid rat secretions, and promptly seek medical care if they have RBF symptoms (4) after contact with rats.
